# Platelet biomarkers for a descending cognitive function: A proteomic approach

**DOI:** 10.1111/acel.13358

**Published:** 2021-05-04

**Authors:** Haitao Yu, Yanchao Liu, Benrong He, Ting He, Chongyang Chen, Jiahua He, Xifei Yang, Jian‐Zhi Wang

**Affiliations:** ^1^ Key Laboratory of Ministry of Education for Neurological Disorders School of Basic Medicine Department of Pathophysiology Tongji Medical College Huazhong University of Science and Technology Wuhan China; ^2^ Key Laboratory of Modern Toxicology of Shenzhen Shenzhen Center for Disease Control and Prevention Shenzhen China; ^3^ School of Physics Huazhong University of Science and Technology Wuhan Hubei China; ^4^ Co‐innovation Center of Neuroregeneration Nantong University Nantong China

**Keywords:** Alzheimer’s disease, machine learning, peripheral biomarkers, platelet, proteomics

## Abstract

Memory loss is the most common clinical sign in Alzheimer's disease (AD); thus, searching for peripheral biomarkers to predict cognitive decline is promising for early diagnosis of AD. As platelets share similarities to neuron biology, it may serve as a peripheral matrix for biomarkers of neurological disorders. Here, we conducted a comprehensive and in‐depth platelet proteomic analysis using TMT‐LC‐MS/MS in the populations with mild cognitive impairment (MCI, MMSE = 18–23), severe cognitive impairments (AD, MMSE = 2–17), and the age‐/sex‐matched normal cognition controls (MMSE = 29–30). A total of 360 differential proteins were detected in MCI and AD patients compared with the controls. These differential proteins were involved in multiple KEGG pathways, including AD, AMP‐activated protein kinase (AMPK) pathway, telomerase RNA localization, platelet activation, and complement activation. By correlation analysis with MMSE score, three positively correlated pathways and two negatively correlated pathways were identified to be closely related to cognitive decline in MCI and AD patients. Partial least squares discriminant analysis (PLS‐DA) showed that changes of nine proteins, including PHB, UQCRH, CD63, GP1BA, FINC, RAP1A, ITPR1/2, and ADAM10 could effectively distinguish the cognitively impaired patients from the controls. Further machine learning analysis revealed that a combination of four decreased platelet proteins, that is, PHB, UQCRH, GP1BA, and FINC, was most promising for predicting cognitive decline in MCI and AD patients. Taken together, our data provide a set of platelet biomarkers for predicting cognitive decline which may be applied for the early screening of AD.

## Graphical abstract

Deep profiling of platelet proteome in healthy aging subjects and MCI/AD cases. Some individual candidate biomarkers in platelets closely reflect the pathophysiological process of AD. The identified novel platelet combination biomarkers can distinguish healthy aging subjects and cognitively impaired subjects.

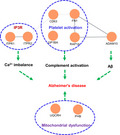

## 1 INTRODUCTION

Alzheimer's disease (AD) is the most common cause of neurodegenerative disorders, and its prevalence is exacerbated by an aging population (Collaborators, 2019). It is estimated that about 47 million people are currently affected by dementia, and the number is expected to reach 131 million by 2050, with appropriate interventions and treatment leading to a reduction in prevalence (Hodson, 2018). The main clinical manifestations of AD patients are memory impairment and cognitive deficits, which make them unable to effectively carry out daily life (Querfurth & LaFerla, 2010). However, the underlying pathology, including amyloid plaque deposition and neurofibrillary tangles, may have occurred before symptoms appear (Hodson, 2018; Jack et al., 2010). Therefore, timely diagnosis, intervention, and treatment are particularly important. However, the diagnosis of AD has not been standardized, and the main diagnostic methods include MRI and PET brain imaging, biochemical analysis of Aβ42/40, and total tau (t‐tau) and phosphorylated tau (p‐tau181) levels in the cerebrospinal fluid (CSF) (Bocchetta et al., 2015; Rice & Bisdas, 2017; Ritchie et al., 2017). Although these diagnostic methods have made significant progress, they are hardly acceptable to the potential patients because these methods are either expensive or invasive. In addition, researchers have paid more attention to the periphery, such as microRNA455‐3p in blood has the potential to serve as a peripheral marker for early diagnosis of AD (Kumar & Reddy, 2018, 2019; Kumar, Vijayan, & Reddy, 2017). Therefore, finding blood biomarkers is of great significance for the early diagnosis of AD.

Platelet, a non‐nuclear fragment from megakaryocytes (Cardigan et al., 2005; Kamath et al., 2001), shares multiple similarities with neuron biology, and it is easily affected by diseases (Akingbade et al., 2018). Once activated, platelets will release a variety of biochemically active factors including cytokines, chemokines, and neurotransmitters (Qureshi et al., 2009). In addition to participating in hemostasis, they also play an important role in the regulation of immunity and inflammation (Gawaz et al., 2005). It has been clearly documented that the specific brain pathology of AD is also reflected in platelets, including an increased membrane fluidity, abnormal cytoskeleton, cytochrome oxidase deficiency, abnormal cytoplasmic calcium flux, abnormal glutamate transporter activity, a decreased phospholipase A2 activity, an increased cytoplasmic protein kinase C level, and an increased oxidative stress level (Kawamoto et al., 2005; Vignini et al., 2007). The brain and platelets contain high concentrations of APP, and during AD, the non‐amyloidogenic pathway enzyme disintegrin and metalloproteinase domain‐containing protein 10 (ADAM10) are down‐regulated and the amyloidogenic pathway enzyme BACE1 is up‐regulated (Colciaghi et al., 2002). The activity of GSK‐3β, which promotes tau hyperphosphorylation and tangle formation in the AD brains, is significantly increased in the platelet of AD and MCI patients (Veitinger et al., 2014). Mao‐B, a mitochondrial protein closely related to mitochondrial damage and neuronal apoptosis, is significantly increased in the platelet of AD patients (Forlenza et al., 2011). In addition, the platelet activation state is positively correlated with the rate of cognitive decline measured by the mini‐mental state examination (MMSE) (Stellos et al., 2010). In short, platelets can reflect the AD‐related pathological events and thus may serve as a perfect peripheral matrix for searching biomarkers to objectively predict AD in early stage.

Proteome has special value in studying disease‐related mechanisms and diagnostic markers, which reveals disease phenotype (Lygirou et al., 2018). Compared with traditional proteomic techniques, TMT‐LC‐MS/MS can capture and quantify proteins in a comprehensive and efficient manner with a smaller sample requirement without offset. Recently, proteomic technology based on mass spectrometry has shown its strong power in the neurological field, such as overall analysis of protein expression level, inter‐molecular correlation, and biomarker screening (Bader et al., 2020; Xiong et al., 2019).

By using TMT‐LC‐MS/MS, we did a comprehensive proteomic analysis in the platelets of MCI and AD patients and as well as the age/sex‐matched control population. We found that multiple pathways, including AD, AMPK signaling, platelet activation, telomerase RNA localization, and complement activation, were remarkably changed in MCI and AD patients. Further PLS‐DA analysis plus machine learning revealed that a combination of decreased proteins PHB, UQCRH, GP1BA, and FINC in platelets could be promising in objectively predict the cognitive decline in MCI and AD patients.

## 2 RESULTS

### 2.1 Common differential proteins and pathways in MCI and AD platelets by whole‐proteome analysis

**FIGURE 1:**
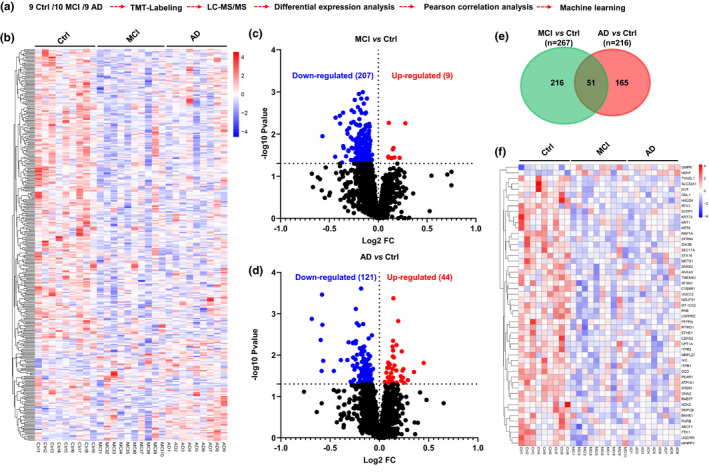
Schematics for study design and the general proteome information. (a) Schematic diagram of platelet TMT‐LC‐MS/MS proteomic operation process. (b) 360 differential proteins were identified in the platelet of MCI and AD patients compared with the Ctrl group, (*p* < 0.05, increased proteins: red; decreased proteins: blue). (c, d) The increased (red) or decreased (blue) level of proteins in MCI vs. Ctrl or AD vs. Ctrl (*p* < 0.05). (e, f) 51 overlapped protein changes in both comparison groups and their relative expression abundance (MCI vs. Ctrl and AD vs. Ctrl)

According to the MMSE score, platelet samples of 10 cases of mild cognitive impairment (MCI; MMSE score 18–23), 9 cases of severe cognitive impairment (AD; MMSE score 2–17), and 9 age/sex‐matched healthy controls (Ctrl; MMSE score 29–30) were collected for proteomic analysis (Figure 1a). The major goals of platelet proteomics data collection and their bioinformatic analysis were set as follows: (a) to analyze the changes of platelet protein profile during the progression of cognitive decline (from normal cognition to MCI to AD); (b) to clarify the biological mechanisms of platelet during the progression of cognitive decline; (c) to find MMSE‐correlated proteins; and finally (d) to identify peripheral diagnostic biomarkers for cognitive impairment.

A total of 2994 platelet proteins were captured by TMT‐LC‐MS/MS proteomics, of which 360 significantly different proteins were identified in MCI and AD patients compared with the Ctrl group (*p* < 0.05) (Figure 1b), relative abundance values were shown in Excel S1. Specifically, 207 differentially expressed (DE) proteins were down‐regulated and 9 were up‐regulated in MCI vs Ctrl (Figure 1c), while 121 DE proteins were down‐regulated and 44 were up‐regulated in AD vs Ctrl (Figure 1d). Moreover, 51 DE proteins were overlapped in both MCI vs Ctrl and AD vs Ctrl (Figure 1e), and all were reduced in MCI and AD (Figure 1f; Excel S2).

**FIGURE 2:**
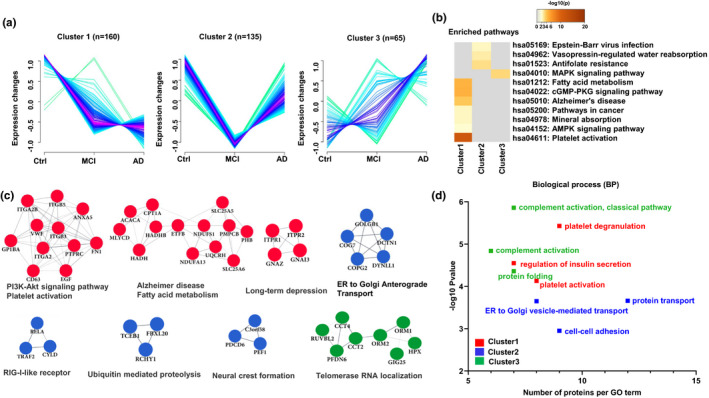
Differential proteins and biological pathways identified in MCI and AD patients compared with normal cognition controls. (a) The protein changes were divided into three clusters according to trends from Ctrl to MCI to AD (each line represents a protein). (b) Pathway enrichment analysis of three cluster proteins with Metascape online analysis (the significantly enriched pathway has been defined as overlap proteins ≥3, *p *< 0.01). (c) Detected PPI modules in clusters. (d) Differential protein enrichment analysis of biological function. Differential protein enrichment analysis of biological function. The red modules, green modules, and green module represent top 3 biological process with –log 10 (*p*‐value) in cluster 1, 2, and 3, respectively

To explore the dynamic changes of platelet proteome during the progression of cognitive decline, we performed cluster and protein–protein interaction (PPI) network analyses in MCI, AD, and the cognitively normal control populations. Proteins in cluster 1 (*n* = 160), including CD63, PHB, UQCRH, ANXA5, and EGF, showed a decreasing tendency from Ctrl to MCI to AD (Figure 2a left). These proteins were enriched in 7 KEGG pathways, including fatty acid metabolism, cGMP‐PKG signaling pathway, AD, pathways in cancer, mineral absorption, AMPK signaling pathway, and platelet activation (Figure 2b). Pathways in cluster 2 (*n* = 135) displayed significant decrease in MCI and increase in AD compared with the normal controls (Figure 2a middle), which were involved in Epstein–Barr virus infections, vasopressin‐regulated water reabsorption, and antifolate resistance (Figure 2b). Proteins in cluster 3 (*n* = 65) showed an increasing tendency from Ctrl to MCI to AD (Figure 2a right), including PPP3CB, STMN1, PTPN7, MAP4 K2, and STK3, all of them were strongly pointed to the MAPK signaling pathway (Figure 2b). By PPI network analysis using MCODE (molecular complex detection) on the differentially expressed proteins, we further defined eight protein interaction modules which supported the identified pathways in the above‐mentioned clusters (Figure 2c). By biological processes analyses of the differential proteins, more comprehensive and detailed biological mechanisms were shown, including regulation of insulin secretion, platelet activation (cluster 1); protein transport, cell–cell adhesion, ER to Golgi vesicle‐mediated transport (cluster 2); and complement activation, protein folding (cluster 3) (Figure 2d).

These whole‐proteome data reveal the total differential proteins and the involved pathways during the progression of cognitive decline in MCI and AD.

### 2.2 Differential platelet proteins or pathways correlated to MMSE score analyzed by Pearson

**FIGURE 3:**
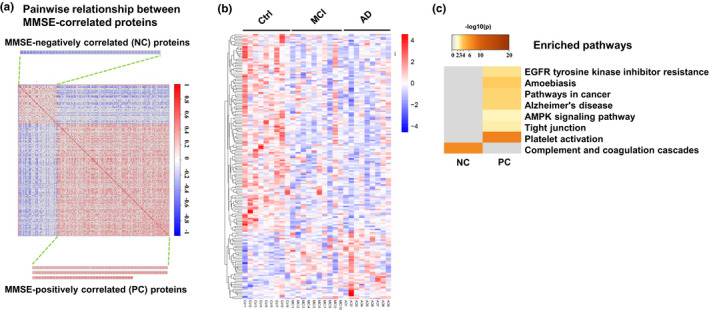
Proteins correlated to MMSE score in platelet proteomics. (a) Negative correlated (NC, blue) and positively correlated (PC, red) proteins to MMSE scores analyzed by Pearson (*p* < 0.05) and ranked according to their coefficients. (b) Heatmap of the relative abundance of all MMSE‐correlated proteins in each sample (increased proteins: red; decreased proteins: blue). (c) Enriched KEGG pathway of all MMSE‐correlated proteins (the significance of the enriched pathway was defined as overlap proteins ≥3, *p* < 0.01)

Mini‐mental state examination (MMSE) score has been wildly used as a subjective measure of cognitive performance. To explore the periphery molecular markers that can objectively predict cognitive impairment, we performed correlation analysis of MMSE score to the entire omics data received from normal Ctrl, MCI, and AD. A total of 173 proteins were identified to be strongly correlated to MMSE score (*p* < 0.05), including 44 negatively correlated (NC) proteins and 129 positively correlated (PC) proteins (Figure 3a‐b), relative abundance values were shown in Excel S3. Interestingly, the NC proteins were selectively enriched in the complement activation pathway (Figure 3c), while the PC proteins were enriched in multiple pathways, including pathways in cancer, AD, AMPK signaling, tight junction, and platelet activation (Figure 3c).

**FIGURE 4:**
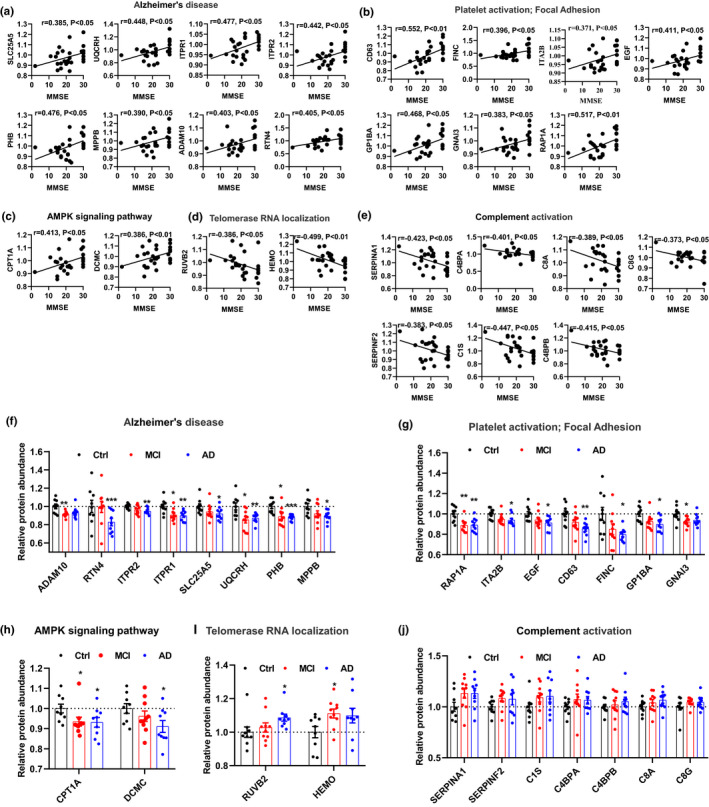
Integration and quantitative analysis of MMSE‐related pathways and DE proteins. (a‐c) KEGG pathway enriched by proteins positively correlated with MMSE score, including Alzheimer's disease (a), platelet activation (b), and AMPK signaling pathway (c). (d, e) KEGG pathway enriched by proteins negatively correlated with MMSE score, including Telomerase RNA localization (d) and complement activation (e). Pearson correlation coefficients (r) and corresponding *p *< 0.05 were displayed at the top of each plots. X‐axis shows MMSE score, y‐axis indicates relative expression abundance of each protein. (f‐j) Dot plots represent the relative expression level of each protein in different samples. Data were presented as mean ± SEM. *p < 0.05, **p < 0.01 and ***p < 0.001 vs. the Ctrl subjects

By further integrating the whole‐proteome differential proteins and MMSE‐correlated proteins, we identified five KEGG pathways closely related to the AD‐related pathological mechanisms, including three PC pathways and two NC pathways (Figure 4a‐e). 19 proteins, involved in the pathways of AD, platelet activation, telomerase RNA localization, and AMPK signaling, were not only differentially expressed proteins but also correlated to MMSE (Figure 4a‐d; f‐i). Specifically, the Aβ‐related protein ADAM10, mitochondrial dysfunction related proteins ADP/ATP translocase 2 (SLC25A5), UQCRH, PHB, mitochondrial‐processing peptidase (MPPB), Ca^2+^ imbalance related proteins ITPR1/2, and endoplasmic reticulum (ER)‐related protein reticulon‐4 (RTN4), in AD pathways (Figure 4a,f); the expression levels of CD63, FINC, ITA2B, EGF, GP1BA, GNAI3, RAP1A (Figure 4b,g), mostly platelet activation‐related molecules [25]; and the level of CPT1A (carnitine O‐palmitoyltransferase 1) and DCMC (Malonyl‐CoA decarboxylase), and as well as AMPK signals pathway (Figure 4c,h) involved in fat metabolism and energy controls (Derdak et al., 2013; Xie et al., 2019); were decreased or decreasing in MCI and AD patients compared with Ctrls (Figure 4a‐c) and a positive correlation MMSE score was shown (Figure 4f‐h). On the other hand, strong and unique enrichment of complement activation pathway (Figure 3c) with a consistent negative correlation to MMSE score was detected in MCI and AD patients (Figure 4e) but the protein levels involved in this pathway was not significantly altered (Figure 4j). Interestingly, the platelet activation, an upstream regulatory pathway of complement activation, was also positively correlated to the MMSE score (Figure 4b), suggesting a peripheral and central connection of the complement pathway that was also observed in the AD brains (Bai et al., 2020).

**FIGURE 5:**
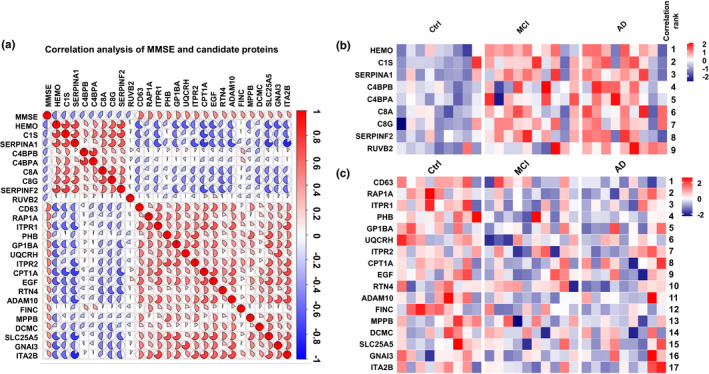
Correlation ranking of candidate protein levels to MMSE score. (a) Negatively correlated (blue) and positively correlated (red) candidate biomarkers (*p* < 0.05) were ranked according to their Pearson correlation coefficients. The ratio of the color shade and the circle represent the degree of correlation. (b, c) The relative abundance of the negatively correlated (b) and positively correlated (c) proteins (brick red represents increased proteins and dark blue represents the decreased proteins)

By further ranking the correlation coefficients of the above 26 platelet candidate proteins to the MMSE score, a complex association was identified (Figure 5a; Excel S4), and the increase or decrease of these candidates was nicely uniformed in each group (Figure 5b). All 26 candidate proteins showed a moderate MMSE correlation (| *r *| =0.371–0.552; Figure 5a; Excel S4). Among them, the reduction of CD63 in platelet showed the strongest correlation with MMSE score (*r *= 0.552, *p *= 0.002; Figure 5a; Excel S4). Interestingly, we also found a close correlation between some of the proteins in the dataset, such as mitochondrial dysfunction related proteins PHB, SLC25A5, MPPB (| *r *| =0.60–0.69); complement activation pathway proteins C1S, SERPINA1, SERPINF2 (| *r *| =0.63–0.75) (Figure 5a; Excel S4). In addition, Aβ‐related protein ADAM10 was found to be strongly correlated with complement activation‐related protein SERPINA1 and platelet activation‐related protein integrin alpha‐IIb (ITA2B) (| *r *| =0.70–0.85) (Figure 5a; Excel S4), suggesting that the three may play a coordinated or antagonistic role in the pathogenesis of AD. These data together demonstrated that these platelet dysregulated proteins imply a complex regulatory network related to cognitive impairment.

### 2.3 Selecting the best combination of platelet biomarkers to predict cognitive decline by machine learning

**FIGURE 6:**
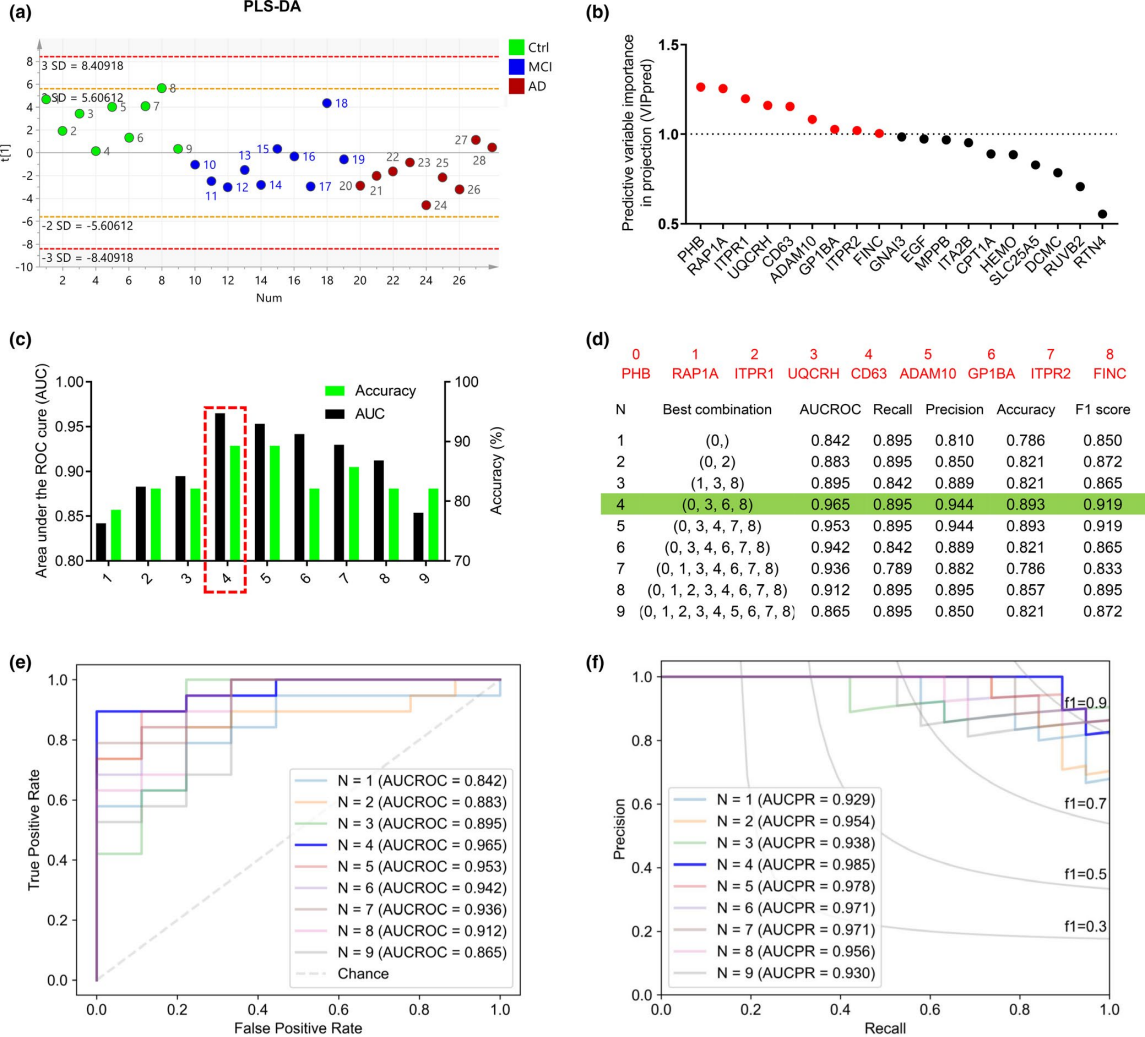
The best combination of platelet biomarkers for cognitive decline identified by machine learning. (a) Discrimination power of the selected 19 candidate platelet proteins from Ctrl to MCI to AD analyzed by PLS‐DA analysis. (b) 9 candidate proteins (VIP>1) were selected by predictive variable importance in projection (VIPpred) analysis (Red bar graph: VIP >1; Green bar graph: VIP <1). (c) The best performing panel based on the area under the receiver operating characteristic (ROC) curve using leave‐one‐out (LOO) algorithm was selected in the training set. Y‐axis‐left; the area under the ROC curve, Y‐axis‐right: accuracy, x‐axis; proteins selected by the LOO algorithm. The red box shows the selected protein with high area under the curve (AUC) and accuracy for the blinded test set. (d) The red numbers represent the corresponding protein. Under each best combination, the corresponding protein and various parameters for evaluating the efficiency of the biomarkers. (e and f) Based on the LOO algorithm, the under the receiver operating characteristic curve (AUCROC) and precision‐recall curve (AUCPR) for each best combination biomarkers. AUCROC was based on true positive rate and false positive rate: True positive rate = [true positive / (true positive +false negative)]; False positive rate = [false positive / (true negative +false negative)]. PRAUC was obtained based on precision and recall: Precision = [true positive / (true positive +false positive)]; Recall = [true positive / (true positive +false negative)]. In addition, F1 score = 2 * (precision * recall) / (precision +recall)

Further machine learning was applied to select the best combination of the biomarkers. Considering the effectiveness of the biomarkers, seven proteins involved in the complement pathway were excluded, and 19 candidate proteins were selected from 26 (Figure 5) for subsequent sample discrimination. Partial least squares discrimination analysis (PLS‐DA) to the selected 19 candidate proteins could nicely distinguish MCI and AD from the Ctrls, though it could not distinguish MCI from AD (Figure 6A). Nine of them, including PHB, RAP1A, ITPR1, UQCRH, CD63, ADAM10, GP1BA, ITPR2, and FINC, were identified as the core contributors to distinguishing normal cognition from the cognitively impaired individuals, and PHB showed the highest differentiation with the predictive variable importance in projection larger than 1 (VIPpred >1) among the nine core candidates (Figure 6B).

To receive the best combination of the platelet biomarkers for predicting cognitive decline, we further analyzed 9 core candidate proteins using leave‐one‐out (LOO) method. This method leaves out one sample at a time as validation set and uses the rest samples as the training set, so that all samples were trained n times and validated n times. By LOO analysis, various specificity and accuracy were observed using different combinations of the 9 biomarkers, and the combination of PHB, UQCRH, GP1BA, and FINC showed the highest specificity with a maximum receiver operating characteristic (ROC) (AUCROC=0.965) and the highest accuracy (89.3%) (Figure 6c‐e). Using this panel of the biomarkers, a generating recall of 0.895, precision of 0.944, F1 score of 0.919, and the largest precision‐recall curve (AUCPR = 0.985) were received (Figure 6d and f). Using this rigorous algorithm, PHB was identified as the most valuable single platelet biomarker to predict cognitive decline, with AUCROC of 0.842, accuracy of 78.6%, recall of 0.895, precision of 0.810, F1 score of 0.850, and AUCPR of 0.929 (Figure 6c‐f).

By using Western blotting to verify the above‐mentioned nine target proteins (PHB, RAP1A, ITPR1, UQCRH, CD63, ADAM10, GP1BA, ITPR2, and FINC), we observed a decreasing trend of the levels of PHB, CD63, GP1BA, and FINC in MCI or/and AD, which was consistent with the proteomic results, but no significant decrease for the other five molecules (Figure S1). The discrepancy might be caused by the method or/and the limited sample size.

Together, the machine leaning further selects out the combination of PHB, UQCRH, GP1BA, and FINC as the best platelet biomarkers for evaluating the cognitive decline in MCI and AD patients.

## 3 DISCUSSION

AD is most common neurodegenerative disorder affecting an increasing number of the populations with old age. As there is no efficient cure for this devastating disease, finding objective periphery biomarkers is extremely important for early diagnosis and drug development of AD.

Recently, proteomics has been widely applied for biomarker studies. With breakthroughs of the technology, we can comprehensively and in‐depth explore the expression changes of the peripheral platelet protein profile for the screening of the periphery biomarkers. Previous studies using platelet samples from AD patients were mostly limited to two‐dimensional gel electrophoresis methods, and only a few to dozens of differential proteins were identified (Gonzalez‐Sanchez et al., 2018; Reumiller et al., 2018), and the highest number of proteins was identified by using LC‐MS/MS (Donovan et al., 2013). Based on the high‐resolution LC‐MS/MS combined with the latest TMT tag technology, our present study had identified 4165 proteins, of which 2994 were effectively captured in each experimental group, providing the most in‐depth platelet proteome changes so far for the cognitive decline in MCI and AD subjects. The high proteome coverage provides a strong guarantee for the subsequent analysis of platelet‐related biological mechanisms in cognitive impairment process and the construction of machine learning models. With such rich proteomic information, three positively correlated modules and two negatively correlated modules with MMSE scores were revealed. The complex regulatory network in platelets, including AD, platelet activation, AMPK signaling, telomerase RNA localization, complement activation, may be closely related to the pathological mechanisms of AD. The resulting candidate proteins could efficiently discriminate MCI and AD from the cognitively normal control population, though it currently could not distinguish MCI from AD.

Integrating the existing brain/CSF proteomics (Bai et al., 2020; Wang et al., 2020), we found some interesting changes in the central and peripheral systems. Consistent with human brain and CSF proteomics, the levels of mitochondrial proteins were decreased and complement‐associated proteins were increased in patients with AD (Bai et al., 2020; Wang et al., 2020). In addition, lipid metabolism‐related proteins were increased in the brain and decreased in platelets of AD (Bai et al., 2020). Importantly, we found that platelet activation, telomerase RNA localization pathway dysregulation was specific in platelets. Platelet and complement activation, calcium imbalance pathways were reported in another platelet proteomics (Donovan et al., 2013).

In addition to Aβ deposition and abnormal tau‐related neurofibrillary tangle formation, AD also includes a variety of pathological changes involving calcium imbalance, autophagy defects, mitochondrial abnormalities, and synaptic damage (Grontvedt et al., 2018). In the current study, we detected multiple enriched proteins in the AD pathways, including mitochondrial dysregulated proteins (PHB, SLC25A5, UQCRH, MPPB), Ca^2+^ flow imbalance (ITPR1, ITPR2), non‐amyloid protein production related proteins ADAM10, endoplasmic reticulum protein RTN4. PHB (inhibin) plays a key role in the regulation of mitochondrial protein homeostasis through the proteolytic machinery m‐AAA protease in the inner mitochondrial membrane (Steglich et al., 1999). PHB also serves as a mitochondrial respiratory chain chaperone protein and the decrease of PHB induces mitochondrial dysfunction and ROS overproduction (Kathiria et al., 2012). MPPB is related to mitochondrial biogenesis (Nagayama et al., 2008), and UQCRH, as a subunit of mitochondrial respiratory chain complex III (Liu et al., 2016), cooperates with SLC25A5 (ADP/ATP transport enzyme 2) to regulate ATP synthesis and transport (Clemencon et al., 2013). Classical neuropathological hallmarks of disease (Aβ and tau) may trigger mitochondrial disturbance, while mitochondrial dysfunction may incite pathology and cognitive deficits (Kerr et al., 2017; Perez Ortiz & Swerdlow, 2019). Inositol 1, 4, 5‐trisphosphate (IP3R) is the most widely expressed calcium ion release channel, which regulates the entry of calcium ions from the endoplasmic reticulum into the cytoplasm (Thillaiappan et al., 2019). Imbalance of calcium ion homeostasis can affect the release of synaptic signal transmitters, mitochondrial dysfunction, increase of ROS production, and ultimately lead to cell death, which can affect amyotrophic lateral sclerosis (ALS), Huntington's disease (HD), AD, and other neurodegenerative diseases (Takada et al., 2017).

Reticulon family members can reduce Aβ generation through negative regulation of β‐secretase (BACE1) (He et al., 2004; Murayama et al., 2006). Several studies have shown that the level of platelet BACE1 increases from the early stage to the late stage of AD (Colciaghi et al., 2004; Marksteiner & Humpel, 2013). Interestingly, the activity of BACE1 in platelets only increases in AD, but does not change in MCI (Bermejo‐Bescos et al., 2013). Moreover, our omics data showed that ADAM10 (α‐secretase) was significantly decreased in MCI, which was consistent with a previous study (Colciaghi et al., 2002). In a cohort study of the elderly in Brazil, it was found that the level of ADAM10 was continually decreased with the degree of cognitive impairment, which has the potential as a diagnostic biomarker for AD (Manzine et al., 2013). Therefore, considering the pathological connection with Aβ deposition and the significant correlation with the clinical symptoms of dementia, ADAM10 and BACE1 could serve as peripheral platelet biomarkers for early diagnosis of AD.

It is well known that patients with AD have significant energy imbalance (Yin et al., 2016), and AMPK signaling pathways play a central role in energy balance (Carling, 2017). We found here that DCMC and CPT1A, involved in lipid metabolism‐related processes regulated by AMPK (Derdak et al., 2013; Xie et al., 2019), were significantly decreased in MCI and AD platelets. In a fatty liver study, pifithrin‐α p‐nitro (PFT) can promote the expression of DCMC by regulating the SIRT1/LKB1/AMPK pathway, and the activity of CPT1A could be stimulated by reducing malonyl‐CoA (mCoA) (Derdak et al., 2013). Studies showed that abnormal lipid metabolism was closely related to AD pathology (Liu et al., 2013; Wong et al., 2017). Cholesterol is an important part of axonal growth, formation, and remodeling (Liu et al., 2013). Therefore, the decreased expression of DCMC and CPT1A in peripheral platelets may be related to the abnormal lipid metabolism in MCI and AD patients. The production of bioactive products of lipid peroxidation leads to continuous platelet activation, which may contribute to amyloid deposition and complications of atherosclerotic thrombosis (Ciabattoni et al., 2007).

Consistent with the previous reports, patients with AD have significant dysregulation in the platelet activation pathway (Akingbade et al., 2018; Veitinger et al., 2014). Epidemiological data show that the increased levels of platelet activation biomarkers, activation of glycoprotein IIb‐IIIa complex and P‐selectin, are significantly related to cognitive decline in AD patients (Stellos et al., 2010). CD63, a member of the four‐transmembrane family, is easily located in the plasma membrane from lysosome during platelet activation (Maduskuie et al., 1998), and cooperates with P‐selectin to promote thrombosis in atherosclerosis (Cha et al., 2003; Yamazaki et al., 2001). Interestingly, we also found that the expression of several proteins (GP1BA, FINC, RAP1A, and VWF) involved in platelet function related to hemostasis and thrombogenesis were decreased in MCI and AD patients. For example, VWF/GP1BA interactions induce platelet activation/adhesion and regulate integrin signaling pathways for hemostasis and thrombosis (Li et al., 2010). FINC (Fibronectin) affects platelet activation by regulating the formation of PF4/heparin complex (Krauel et al., 2019). RAP1A and RAP1B, important components of RAP GTPase, identify injured sites and as important switches for platelet adhesion and activation to ensure vascular integrity (Stefanini & Bergmeier, 2019). The collective reduction of the platelet activation‐related proteins may affect hemostasis and maintain normal vascular function, which is consistent with the vascular risk factors of AD patients, such as diabetes, hypertension, atherosclerosis (Casserly & Topol, 2004; Helzner et al., 2009; Huo et al., 2003).

Our proteome data showed that the proteins negatively correlated with MMSE scores had strong enrichment in the complement activation pathway, suggesting a strong complement inflammatory response in the peripheral system; however, only slight increase of the complement activation pathway proteins (SERPINA1, C4BPA, C8A, C8G, SERPINF2, C1S, and C4BPB) were detected in MCI and AD patients. Recently, the complement pathway has attracted great attention, which is involved in the regulation of microglial synaptic pruning in the early stage of AD (Hong et al., 2016), and C1q‐blocking antibody reverses synaptic damage in Tau‐301S mice (Dejanovic et al., 2018). Brain proteomics also revealed that the complement pathway (C1R, C1S, C3, C4A, and C4B) was activated during progression of MCI into AD (Bai et al., 2020). Our data may be a good addition to illustrate the synchronous activation of the complement pathway in the peripheral and central systems.

Based on the nine candidate proteins identified from whole‐proteome and MMSE correlation, we conducted further machine learning. After twenty‐eight rounds of training and testing, the strict LOO algorithm revealed that combination of platelet PHB, UQCRH, GP1BA, FINC could most accurately identify the cognitive decline in MCI and AD patients. Interestingly, the four molecules identified by the machine learning algorithm represent two important pathological processes, that is, the mitochondrial dysfunction (PHB, UQCRH) and platelet activation (GP1BA, FINC).

In summary, such in‐depth and comprehensive analysis of peripheral platelet protein expression profiles of MCI and AD patients has given us new understanding of the role of platelets in AD. Bioinformatics analysis revealed that the linkage effect between peripheral and AD reflected by platelet omics involved platelet activation, complement pathway activation, mitochondrial dysfunction, calcium ion imbalance, and APP metabolic abnormality. Machine learning identified distinctive cognitive impairment‐platelet combination biomarkers (PHB, UQCRH, GP1BA, and FINC). Altogether, the exploration of platelet proteomics is novel and a great supplement to understanding the peripheral changes of AD, and platelet combination biomarkers have great application potential in precision medicine for AD.

## 4 EXPERIMENTAL PROCEDURES

### 4.1 Participants’ information

According to mini‐mental state examination (MMSE) score (Folstein et al., 1975) and National Institute on Aging and the Alzheimer's Association Guidelines (Albert et al., 2011), we recruited 28 Han People and divided them into three groups: with mild cognitive impairment (10 MCI, MMSE = 18–23), with severe cognitive impairment (9 AD, MMSE = 2–17) and the age‐/sex‐matched normal cognition controls (9 Ctrl, MMSE = 29–30) (Table 1). Any cases with head trauma, brain tumor, epilepsy, transient ischemic attack, coma, drug abuse, alcohol addiction, depression, schizophrenia, and other psychiatric disorders were excluded in all the samples. The influence of confounding factors such as apolipoprotein E (APOE), Aβ1‐42/1‐40, diabetes, hypertension, hyperlipidemia, and coronary heart disease (CHD) were considered comprehensively (Table 1).

**TABLE 1:** Information for patients and the age‐/sex‐matched controls

Characteristic	Ctrls (*n* = 9)	MCI (*n* = 10)	AD (*n* = 9)	*p‐value*
Age, mean (*SD*), year	72.67 (2.60)	72.50 (2.46)	73.11 (5.21)	0.931
MMSE (*SD*)	29.89 (0.33)	21.00 (1.56)	13.56 (4.69)	<0.001
Sex (male, female)	4 M, 5F	4 M, 6F	4 M, 5F	0.974
Diabetes mellitus, *n*	0	0	0	>0.999
Hypertension, *n*	4	7	5	0.528
Coronary heart disease (CHD), *n*	2	1	1	0.709
Cerebral apoplexy, *n*	0	2	4	0.071
APOE ε2 (+), *n* (%)	2 (22.2%)	1 (10.0%)	1 (11.1%)	0.685
APOE ε3 (+), *n* (%)	9 (100%)	9 (90.0%)	8 (88.9%)	0.467
APOE ε4 (+), n (%)	0 (0.0%)	2 (20.0%)	2 (22.2%)	0.364
Aβ1‐40 (*SD*)	335.6 (299.8)	307.1 (299.6)	502.2 (315.5)	0.347
Aβ1‐42 (*SD*)	67.88 (26.46)	59.87 (42.65)	61.16 (22.27)	0.849
Aβ1‐42/1‐40 (*SD*)	1.02 (2.07)	1.19 (2.11)	0.29 (0.39)	0.504

Abbreviations: AD: severe cognitive impairment; APOE: apolipoprotein E; Ctrl: normal cognition controls group; MCI: mild cognitive impairment; MMSE: the Minimum‐mental State Examination.

The study was approved by the Tongji Medical School Ethics Committee, complies with the Helsinki Declaration II, and includes written informed consent from all participants.

### 4.2 Sample preparation

The fresh blood stored in the anticoagulant tube was centrifuged at 200 g for 20 min to remove the rich red blood cells and white blood cells from the plasma, and 2/3 of the platelet‐rich supernatant was taken to the new centrifuge tube. Next, the platelet‐rich plasma was centrifuged at 120 g for 6 min to remove residual white blood cells and centrifuged at 1,500 g for 10 min to obtain relatively pure platelet precipitate. Further, the platelet precipitate was washed with tyrode's solution (143.0 mM NaCl, 5.4 mM KCl, 0.25 mM NaH2PO4, 1.8 mM CaCl2, 0.5 mM MgCl2, 5.0 mM HEPES, pH 7.4; Solarbio, T1420, Beijing, China) and centrifuged at 120 g for 4 to obtain purified platelet samples and stored at −80°C.

Platelet samples were added with lysis buffer (8 M urea, pH 8.0, 1 cocktail, 1 mM PMSF) and completely lysed by ultrasound (120 s, 4 s on and 6 s off). After lysis of the ice for 30 minutes, the samples were centrifuged at 12,000 g for 10 minutes to obtain protein solution.

### 4.3 Tandem mass tag (TMT) labeling

We performed a proteomic analysis of a large sample size (*n* = 9–10), and each sample corresponds to a TMT label (ThermoFisher 90406). Each sample was digested with mass spectrometric trypsin (Promega, V5072) into peptides and then labeled with TMT. Each labeled peptides was mixed into a group of 10 different labeled samples, which were divided into 15 components by high‐performance liquid chromatography (HPLC) for subsequent experiments.

### 4.4 Data collection of TMT‐labeled peptides using LC‐MS/MS

The dried components were dissolved in 0.1% formic acid (FA), and captured with a silica gel capillary column filled with C18 resin (Varian, Lexington, MA, USA) for subsequent Q Exactive (Thermo Scientific, NJ, USA) mass spectrometer analysis. Full scan in Orbitrap mass analyzer in data‐dependent acquisition (DDA) mode, the specific parameters are set as follows: 400–1, 800 m/z, 70000 resolution; MS/MS scans (100−1,800 m/z). Using Proteome Discoverer 2.1 software (Thermo Scientific) to retrieve MS/MS data according to Uniport‐human database (2020–05). The searching parameters were modified on the previous research settings (Xu et al., 2019).

### 4.5 Bioinformatics analysis

After we normalized and filled the data on the Perseus platform, we used the *t* test method to calculate the *p*‐value of the protein abundance of log2‐transformed between each comparison group (ctrl vs MCI; ctrl vs AD; MCI vs AD) (Bereczki et al., 2018). Proteins with *p *< 0.05 were defined as differentially expressed in the comparison group. R studio (v. 0.99.489) and heatmap gplots package were used for cluster analysis and heatmap drawing. Volcano plot and heatmap were performed using GraphPad Prism 8.00. Pathway and biological function enrichment of statistical clusters was performed using Metascape (http://metascape.org), WEB‐based GEne SeT AnaLysis Toolkit (http://www.webgestalt.org) and DAVID version 6.7 (https://david‐d.ncifcrf.gov/). Cytoscape 3.6.1 and STRING (v10; https://string‐db.org/) plug‐in were used for visual analysis of protein–protein interaction (PPI) network. We use 'Wu Kong' platform (https://www.omicsolution.com/wkomics/main/) for relative Pearson analysis.

### 4.6 Machine learning

SIMCA (version 14.0) software was used for partial least squares discrimination analysis (PLS‐DA). The protein of predictive variable importance in projection larger than 1 (VIPpred >1) was considered to be meaningful for sample discrimination. Further leave‐one‐out (LOO) cross validation was applied to select candidate biomarkers. The samples were trained and evaluated in a leave‐one‐out manner using scikit‐learn python package. Logistic regression was chosen as the classifier with {\rm solver='liblinear’} and {\rm class weight='balanced’}. Specifically, twenty‐eight cross‐validations of the proteome samples (Ctrl = 9, MCI = 10, AD = 9) were performed, in which twenty‐seven were randomly attributed into the training set, and one was in the validating set for each analysis. By twenty‐eight cycles, all the samples had been validated. Based on the LOO algorithm, the under the receiver operating characteristic curve (AUCROC) and precision‐recall curve (AUCPR) for each best combination biomarkers. AUCROC was based on true positive rate and false positive rate: True positive rate = [true positive / (true positive +false negative)]; False positive rate = [false positive / (true negative +false negative)]. PRAUC was obtained based on precision and recall: Precision = [true positive / (true positive +false positive)]; Recall = [true positive / (true positive +false negative)]. In addition, F1 score=2 * (precision * recall) / (precision +recall).

### 4.7 Western blot analysis

The primary antibodies, anti‐ITPR1 (1:2000, Affinity, DF3000), anti‐ITPR2 (1:2000, Affinity, DF13336), anti‐FINC (1:2000, Abcam, ab45688), anti‐GP1BA (1:2000, Abcam, ab134087), anti‐ADAM10 (1:2000, Affinity, AF5294), anti‐CD63 (1:2000, Abcam, ab134045), anti‐PHB (1:3000, Abcam, ab75766), anti‐RAP1A (1:2000, Affinity, DF6157), anti‐UQCRH (1:1000, Abcam, ab154803) were added and incubated on ice overnight. After washing with TBST, the membranes were incubated with anti‐rabbit or anti‐mouse IgG HRPs (Thermo Fisher Scientific, 1:3000) for 50 min at room temperature. Then the membranes were treated with enhanced chemiluminescence (ECL) reagents from an ECL kit (Pierce, Thermo Scientific) for exposure.

### 4.8 Statistical analysis

Statistical analysis was performed by SPSS 24.0 software (Statistical Program for Social Sciences Inc., Chicago, IL, USA). We used one‐way variance analysis (ANOVA) to evaluate the statistical differences for the population information and Western blotting results, and the student's t test to compare the proteomic results of two groups. *p* < 0.05 was considered to be significant, and the data were expressed as mean ± *SEM*.

## CONFLICT OF INTEREST

The authors declare that they have no conflict of interest to disclose.

## AUTHORS’ CONTRIBUTIONS

Experimental design: HY, XY, and JW; recruitment of subjects and sample collection: HY, YL, BH, and TH; experimental methods: HY, YL, CC, XY, and JW; data analysis: HY, YL, CC, and JH; manuscript writing: HY, XY, and JW.

## Supporting information

Fig S1

## Data Availability

All data used to support the findings of this study are included within the article. Raw data used to generate the figures are available from the corresponding author upon request.
