# Supplementary figures and images for: Platelet biomarkers for a descending cognitive function: A proteomic approach
Source: Aging Cell. 2021 May 4;20(5):e13358. doi: 10.1111/acel.13358 (PMC8135080; doi:10.1111/acel.13358)

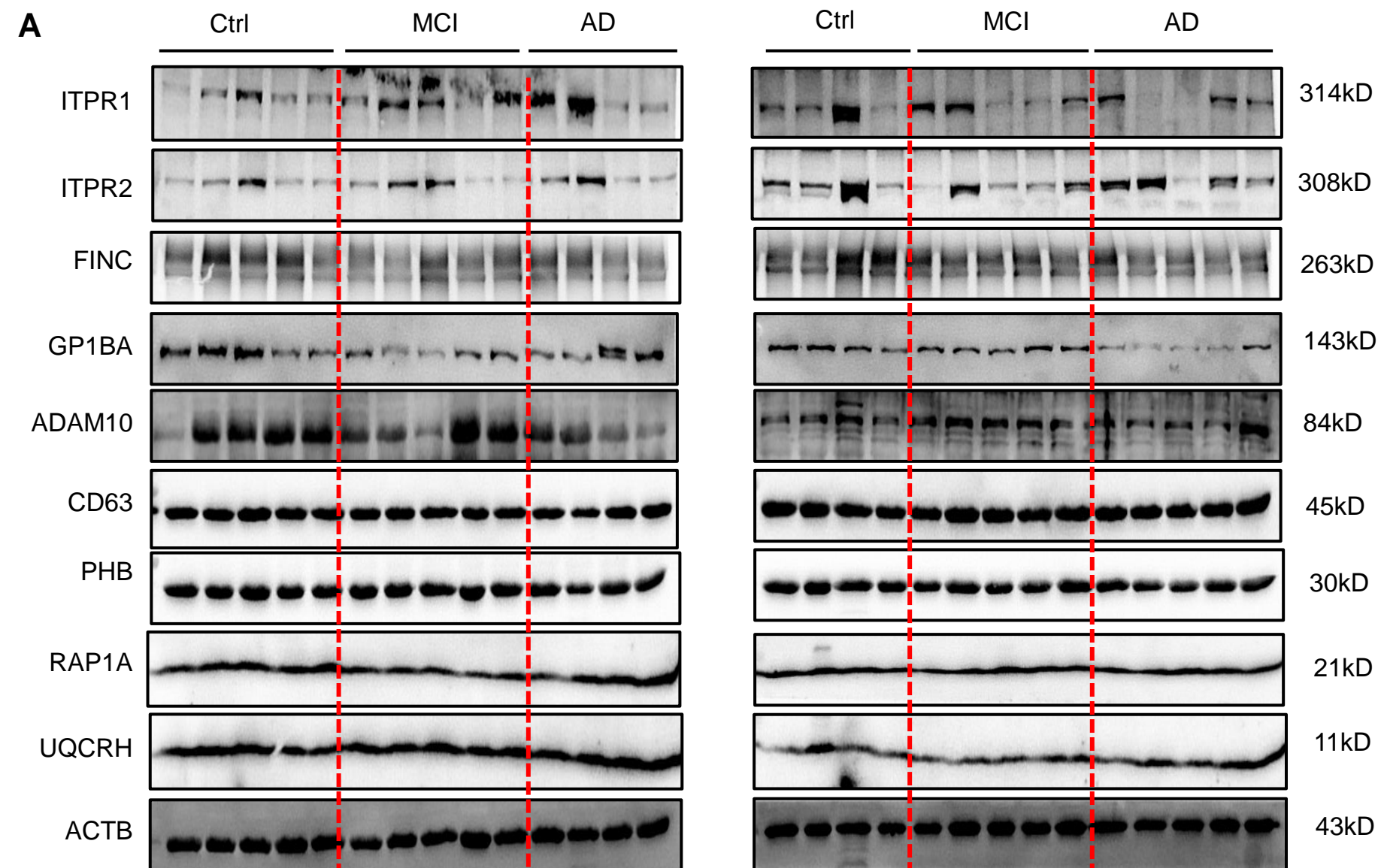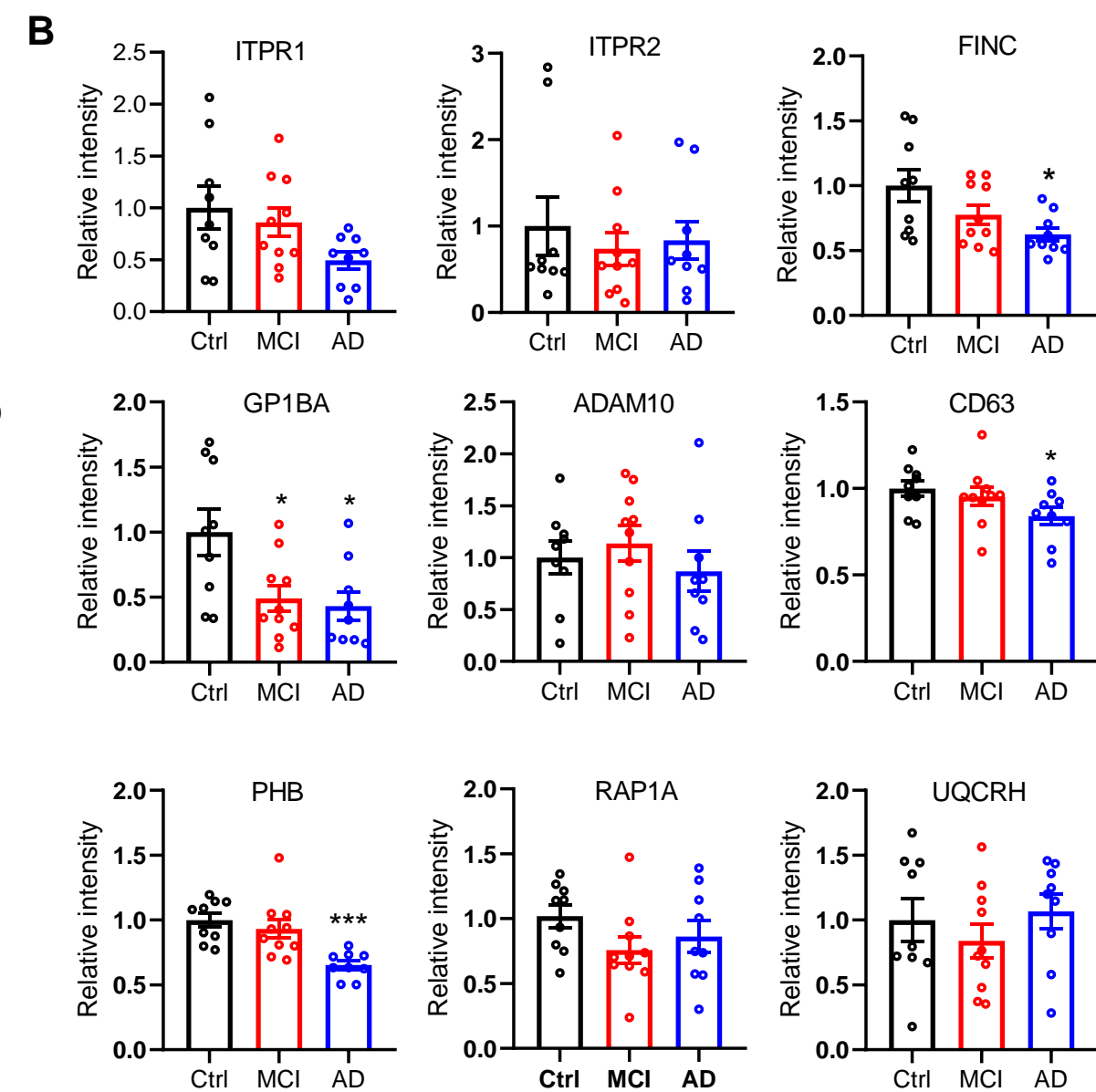

Supplementary figure 1

Yu haitao et al., 2021

Supplement: Supplementary file 1 — Fig S1 [file ACEL-20-e13358-s001.pdf]
